# Towards an explanatory framework for national level maternal health policy agenda item evolution in Ghana: an embedded case study

**DOI:** 10.1186/s12961-018-0354-5

**Published:** 2018-08-03

**Authors:** Augustina Koduah, Irene Akua Agyepong, Han van Dijk

**Affiliations:** 1 University of Ghana, School of Pharmacy, P. O. Box LG43, Legon, Accra, Ghana; 2 Ghana Health Service, Research and Development Division, Dodowa Health Research Center, Dodowa, Ghana; 3 Sociology of Development and Change, Wageningen University & Research Centre, Wageningen, Netherlands

**Keywords:** Decision pathway, Ghana, Maternal health, Policy actors, Policy agenda, Policy evolution, Policy fate, Power

## Abstract

**Background:**

Understanding decision-making processes that influence the fate of items on the health policy agenda at national level in low- and middle-income countries is important because of the implications for programmes and outcomes. This paper seeks to advance our understanding of these processes by asking how and why maternal health policy agenda items have fared in Ghana between 1963 and 2014.

**Methods:**

The study design was a single case study of maternal health agenda evolution once on a decision pathway in Ghana, with three different agenda items as sub-units of analysis (fee exemptions for maternal health, free family planning and primary maternal health as part of a per capita provider payment system). Data analysis involved chronologically reconstructing how maternal health policy items evolved over time.

**Results:**

The fate of national level maternal health policy items was heavily influenced by how stakeholders (bureaucrats, professional bodies, general public and developmental partners) exercised power to put forward and advocate for specific ideas through processes of issues framing within a changing political and socioeconomic context. The evolution and fate of an agenda item once on a decision pathway involved an iterative process of interacting drivers shaping decisions through cycles of ‘active’ and ‘static’ pathways. Items could move from ‘active’ to ‘static’ pathways, depending on changing context and actor positions. Items that pursued the ‘static’ pathway in a particular cycle fell into obscurity by a process that could be described as a form of ‘no decision made’ in that an explicit decision was not taken to drop the item, but neither was any policy content agreed. Low political interest was exhibited and attempts to bring the item back into active decision-making were made by actors mainly in the bureaucratic arena seeking and struggling (unsuccessfully) to obtain financial and institutional support. Policy items that pursued ‘active’ pathways showed opposite characteristics and generally moved beyond agenda into formulation and implementation.

**Conclusion:**

Policy change requires sustaining policy agenda items into formulation and implementation. To do this, change agents need to understand and work within the relevant context, stakeholder interests, power, ideas and framing of issues.

## Background

How and why national level health policy agenda items evolve and fare once on a decision agenda is an important enquiry for both scholars and policy-makers who seek to understand and inform public policy development and implementation. By decision agenda we refer to a list of items within the government policy agenda that are up for an active decision [1]. They could also be described as having entered a ‘decision pathway’. Decision-making processes that maintain or move policy items into active decision-making are of great importance because they influence which concrete measures are prioritised and potentially moved on into implementation.

A key factor in policy item evolution and fate once it enters active decision-making is how policy advocates and advisers constantly frame ideas within a given context to appeal to powerful stakeholders and policy elites. Policy advocates and advisers launch campaigns to gain political attention and support for an issue as a way for the item to gain entry to the decision agenda and then mobilise support once the issue is actively being considered [2]. Policy issues are constantly revised to shape the policy agenda [3], and such revising is critical for public policy development and implementation. Despite the importance of understanding how health policy items evolve and fare once on the decision agenda, this aspect of policy studies has received relatively little attention in low- and middle-income country (LMIC) settings, with most studies being from high-income countries (for example, [1, 4–6]). The limited work from LMICs has moreover focused on how issues gained prominence and access onto the agenda [7] rather than their fate in decision-making once on the agenda. This is a phase of the policy process that could be considered as the overlap area between agenda setting and policy formulation.

To advance our understanding on this part of the process, we investigated how and why maternal health policy items evolved and fared once on the decision agenda in Ghana. Our work contributes to the relatively limited literature on the policy process in LMICs, providing insights on policy formulation processes and, specifically, on the dynamics and drivers of agenda item evolution and fate in the Ghanaian context.

This paper draws on empirical data from three contrasting case studies of maternal health policy items and their evolution and fate over time in Ghana. The policy items were fee exemptions for maternal healthcare (antenatal, deliveries and postnatal), free family planning as part of national health insurance scheme (NHIS) benefit package and primary maternal healthcare service provision as part of the per capita provider payment system benefit package. Based on synthesis of findings of the three cases, we theorise that decision agenda item evolution and fate is influenced by a complex interaction of changing context, policy actors, issue framing, and policy content and ideas. This includes how national and international policy actors use various forms of power to put forward and advocate for specific ideas through processes of issues framing to influence perceptions of policy content within changing and therefore fluid national and international contexts.

## Methods

Our study design was a holistic (single unit of analysis) case study of national level maternal health policy item evolution and fate, with embedded sub-units of analysis [8]. The context was Ghana, a lower middle-income country in west sub-Saharan Africa. The three embedded sub-units of analysis (cases) were purposively selected for several reasons. Firstly, as noted by Sabatier [9], a decade and over is a long enough period to observe policy change. We therefore wanted to have at least one sub-unit of analysis that enabled us to study a contemporary maternal health policy agenda item that had roots of at least a decade or more. Secondly, the cases had to be maternal health policy items that were still active so that direct observation of ongoing policy actor’s interactions during contemporary decision-making processes was possible. Finally, the ability to go back to documentation of initial decisions that placed the policy items on the decision agenda, whether as written memos, legislation or publicly made statements by national level policy elites, was also critical.

It was difficult to find maternal health policy items that met the criteria of still being active in contemporary times but with several decades of historical evolution and good enough records to track the initial decision criteria and subsequent decisions. The one case we found that met all three criteria was maternal health fee exemptions. Our other two policy items (primary care maternal health services as part of the NHIS per capita benefit package and family planning as part of NHIS benefit package) had less than a decade of evolution, though the issues were still ongoing and direct observations and documentation was possible. This was a weakness since the first case differs from the two other cases in terms of the time periods we studied. Replication logic is an important mechanism for testing ideas from one case against subsequent cases. Our first case (free maternal care) goes back several decades, covering different political systems from the one party socialist rule of Kwame Nkrumah in 1963, when it first appears on the decision agenda, through several military governments interspersed with failed attempts at multi-party democracy, into the period of the fourth republic that started in 1992. Our next two cases do not. Primary care maternal health services as part of the NHIS per capita benefit package goes back to 2010 and family planning as part of the NHIS benefit package goes back to 2008. We have however kept all three cases in the analysis because we think apart from this difference, they still share enough commonality in our other criteria to provide useful insights into our study question of the how and why of the evolution and fate of maternal health agenda items over time. It also provides the opportunity to explore contrasts between the case that had evolved over several decades and the two cases that had evolved for less than a decade.

AK (one of the authors) collected data between May 2012 and August 2014 using document review, qualitative interviews, participant and non-participant observations. Document review was used to map national level maternal health policy content, identify policy actors’ roles, actions and interactions in maternal health policy processes and to triangulate and validate evidence from other data sources. Documents reviewed (summarised in Table 1) included government legislation such as Laws and Acts of Parliament, as well as health sector reports, meeting records and media reports. The data extracted from the documents were grouped and cross analysed, as suggested by Robson [10], to obtain rigorous and valid inferences.

**Table 1:** Documents reviewed across the case studies

Document	Year
Laws	
Ghana Health Service and Teaching Hospital Act 525	1996
Hospital Fees Act 387	1971
Hospital Fees Ordinance CAP 82 (1897)	1942
Hospital Fees Decree, National Liberation Council Decree 360	1969
Hospital Fees Regulations, Legislative Instrument 1277, 1313	1983, 1985
National Health Insurance Act 650, 852	2003, 2012
National Health Insurance Regulations, Legislative Instrument 1809	2004
The 1992 Constitution of Ghana	1992
Reports	
Health sector aide memoire	2001–2013
Aligning exemption policy and practice with poverty reduction goals	2003
Annual programme of work	2002–2014
Independent review of the annual programme of work	2001–2013
Inter-agency leadership committee report (April)	2009
Health sector medium-term development plan	2002–2006, 2007–2011, 2010–2013
Pro-poor agenda	2004
Report of the committee appointed to investigate hospital fees	1970
Review of the exemption policy	2006
Health sector response to maternal mortality	2004
Meeting records	
Health sector working group meetings	12 January 2012, 9 February 2012, 3 May 2012, 7 June 2012, 5 July 2012
Inter-agency leadership committee meeting	9 March 2011, 1 December 2011, 10 April 2012
Provider payment mechanism technical subcommittee	2010–2012
Media articles	
Ghana News Agency	2008–2014
Ghanaian Daily Graphic	1957–2014

Across the three embedded case studies, 52 respondents were interviewed (summarised in Table 2) to obtain varied perspectives. They included national level policy-makers as well as implementers. For confidentiality, names and positions of respondents are not stated.

**Table 2:** List of respondents

Respondent type	Number
Government	
Ashanti Regional Health Directorate	2
Ghana Health Service headquarters	5
Minister of Health	1
Ministry of Health former staff	3
Ministry of Health	10
National Health Insurance Authority	4
Provider payment mechanism technical sub-committee	2
Public health facility service provider in Ashanti region	4
Non-government	
Christian Health Association of Ghana	1
Coalition of non-government organisation in health (National and Ashanti region representative)	2
Donors	8
Health professional bodies	5
Opposition politician (former Minister of Health)	1
Private not-for-profit service provider	1
Private self-financing service providers	3

The interviews, lasting on average 1 hour, were conducted using a flexible questioning format through face-to-face, telephone and Skype. Interviews were tape recorded for later transcription. However, where permission was not granted, notes were taken maintaining as far as possible the respondent’s precise words and with subsequent verification. Data was also collected by emailing questions to respondents who preferred to participate this way and email back their responses.

AK was attached to the Policy Analysis unit of the Policy, Planning, Monitoring and Evaluation division of the Ministry of Health (MOH), Ghana, as a student researcher for the duration of the fieldwork. Participant and non-participant observations of ongoing policy discussions, including those related to the three maternal health policy agenda items that formed the sub-units of analysis, were used, as noted by Patton [11], to better understand and capture the context in which people interact. The meetings attended, with an average discussion period of 4 hours, are listed in Table 3. Observing and documenting actors interactions and discussions from start to end of these meetings allowed us to draw inference from ongoing health policy discussions to interpret retrospective data from documents and interviews. These meetings included ‘behind the scenes’ negotiations such as the pre-health summit meeting as well as ‘open’ meetings such as multi-stakeholder health summit meeting.

**Table 3:** Meetings observed and documented for the case studies

Meetings	Dates
Health sector business meeting	17 August 2012
	20 November 2012
	2 May 2013
	20–21 November 2014
Capitation policy evaluation	12 February 2013
DFID meeting on health sector budget support	15 November 2012
Free family planning committee meeting	13 December 2012
Health sector working group meetings	5 July 2012
	6 September 2012
	7 February 2013
	20 March 2014
Inter-agency performance review meeting	16–17 August 2012
	12–13 September 2013
	4–5 April 2013
	20–21 August 2014
MDG Acceleration Framework (MAF) regional (Central & Western) planning meetings	8–12 October 2012
MAF teaching hospitals and training institutions planning meeting	3–5 January 2013
MAF national monitoring and evaluation	12 October 2012
Ministry of Health (MOH) budget committee meeting	5 September 2012
MOH budget hearing at the Ministry of Finance and Economic Planning	20 September 2012
MOH internal review meeting	7 August 2012 21 March 2013
Monitoring visit to Ashanti region; series of meetings	6–9 November 2012
Multi-stakeholder health summit	29–30 April 2013
National workshop with accountability framework with special reference towards women and children’s health meeting	3–4 October 2012
National Health Insurance Authority stakeholder meeting	21–22 December 2012
Policy, planning monitoring and evaluation (PPME) general meeting	25 June 2012
	30 August 2012
PPME unit heads meeting	10, 23 July 2012
	13 August 2012
	4, 10 September 2012
	15, 22 October 2012
	12, 19 November 2012
Pre-budget review meeting	12 September 2012
Pre-business meeting	16 November 2012
Pre-health summit meeting	19 April 2013
Provider payment mechanism technical subcommittee	29 August 2014
Stakeholder meeting on institutional mortality	27–28 March 2014

Initial findings were presented to policy actors individually as well as fora such as the provider payment mechanism technical subcommittee meeting and the Policy, Planning, Monitoring and Evaluation unit heads meetings for discussions, comments and critique. The discussions of preliminary findings and conclusions with respondents and other policy actors allowed for further clarifications of issues less understood by the researcher and validation of issues with conflicting findings such as specific decision timelines.

Data from the interviews, observations and document reviews were tabulated and systematically grouped based on the individual cases. The initial analysis involved mapping out patterns and themes. The emerging themes and patterns from the data were coded. Further analysis involved chronologically reconstructing how the maternal health policy agenda items evolved over time. We acknowledge the challenge involved in restructuring exact sequence of events. To minimise this, we drew from research observation notes and IAA’s experience as a participant in the health sector decision-making processes first as a district and then a regional health director from 2000 to 2013. Further interpretations were therefore made based on our observations and experience of how policy actors constantly frame ideas within a specific context to gain attention and support. The analyses were synthesised to reconstruct how maternal health policy items evolved on decision agenda and why.

The individual cases were further cross analysed, as suggested by Yin [8], to identify core consistencies, contrasts, meanings and pathways through which the three maternal health policy items evolved over time once they appeared on the decision agenda. Based on findings of the cross analysis, we propose an explanatory framework of how and why maternal health policy items evolved on decision agenda in Ghana.

### Definition and labelling

The term ‘decision agenda’ refers to a list of items within the government agenda that are up for an active decision [1]. Once an item enters the decision agenda it evolves through decision-making processes to a particular fate. An agenda item can go through one or more decision-making processes with a given fate as the ‘outcome’ of each process. We defined an agenda item fate as the record of a decision (or no decision) taken on the item at the conclusions of any particular deliberation or decision-making process related to the item. This decision (or no decision) could be at the end of a single meeting or at the end of several meetings, but would be clearly documented as a ‘decision’ on the item. Where no decision was documented on the item and no records of any further deliberation on the item or any action taken in relation to it was evident over time, we treated it as having disappeared by a static pathway of ‘no decision made’. This is included in the fate category of ‘decision agenda item static pathway’ with fate as disappearance. We defined ‘agenda item disappearance by active pathway’ as an item that is mentioned at least once in records and documentation of policy processes and discussions with an active decision to drop it from the decision agenda. We defined ‘agenda item reappearance by active pathway’ as a decision agenda item that had disappeared from records and documentation of policy processes, discussions and decisions, and then reappears at a later period unchanged. We defined ‘agenda item reset by active pathway’ as a decision agenda item that reappears but in a modified form from its statement at the time of first appearance or after disappearing and reappearing. We defined ‘agenda item progress unchanged by active pathway’ as a decision agenda item moving forward into policy formulation and into implementation in its original form.

This study forms part of a larger study, ‘Accelerating progress towards attainment of Millennium Development Goals 4 and 5 in Ghana through basic health systems function strengthening’, for which ethical approval was granted by the Ghana Health Service Ethical Review Committee and the School of Social Science Research Assessment Committee of Wageningen University and Research Centre. Informed consent was obtained from all respondents, and respondent’s anonymity was maintained and protected using codes as labels during the study.

We present the explanatory framework developed from the cross synthesis first, followed by illustration with examples from the three embedded cases. Detailed findings from each of the three embedded cases are provided elsewhere [12].

## Results

### Overview of the maternal health decision agenda item evolution and fate explanatory framework

**Fig. 1:**
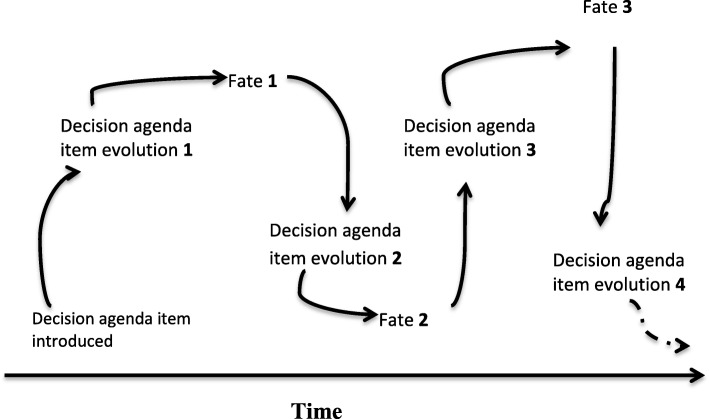
The repeating cycles over time of decision agenda item evolution and fate: an iterative process

**Fig. 2:**
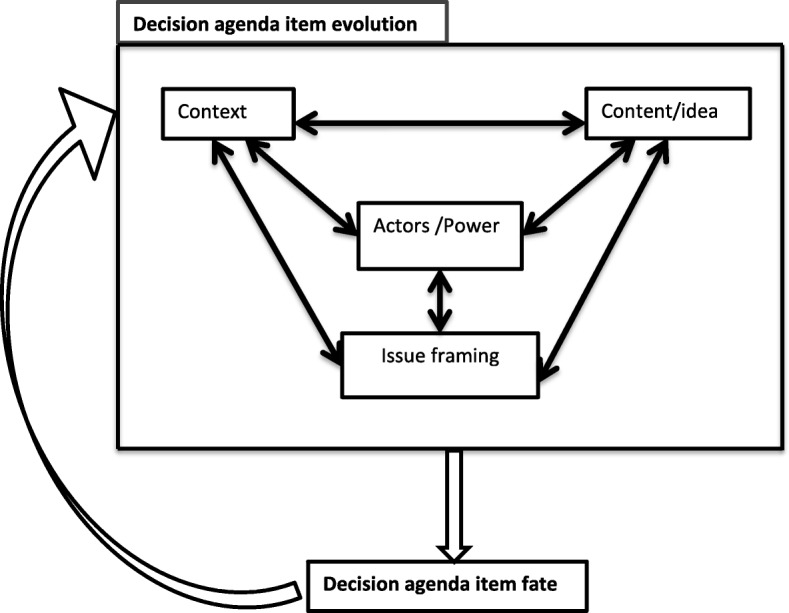
Factors affecting decision agenda item evolution and fate in each cycle

Repeating cycles over time are part of the explanatory framework for maternal health agenda item evolution and fate that we theorise based on our data as illustrated in Fig. 1. The evolution and fate processes are iterative in the sense that the fate in one cycle can, at another time, feed into a new evolution and fate process (Fig. 1). Decision agenda item evolution is driven by factors related to context, policy content and ideas, national and international actors and their use of power, and processes of issue framing (Fig. 2). These factors interact and influence each other in a complex rather than linear manner. Our observation of context, content (issue definition, framing), and actor interactions in processes that influence government agenda item evolution and fate reaffirm the enduring validity and relevance of Walt and Gilson’s policy triangle framework [13]. It is important to note that the term ‘fate’ refers to a particular decision-making cycle in relation to the agenda item rather than a final ‘outcome’. A new cycle may subsequently start with the agenda item that maintains or changes its fate.

### Decision agenda item evolution factors

#### Context

Individual (micro), institutional (meso), national and international (macro) contextual factors all shaped the maternal health policies we studied. Individual or micro contextual factors included ideological predispositions, technical expertise of bureaucrats, professional expertise and interest. The institutional or meso context included administrative capacities of institutions, institutional experiences of previously implemented policies, and institutionalised policy-making processes and structures within the health sector. National or macro contextual factors included interest of professional bodies and general population, national strategic plans and policies, government financial support for implementation, politics, and historical experiences. International (also macro) contextual factors included international agendas such as Millennium Development Goals (MDGs), interest of international activists and advocates, political economy of international relations, austerity measures, and loan and grant conditionalities. Political context, such as change in government, election year, public unrest, demonstrations and social pressures, particularly played a pivotal role in maternal health policies evolution. Short-lived governments and several coups with the incursion of military in national politics characterised the first three and half decades after Ghana’s independence (1957). The last two and a half decades (since 1992) have been characterised by stable multiparty democracy of the fourth republic with elections every 4 years and peaceful transitions of power to an opposition party in several instances. The replacement of the instability of frequent changes in government through coups with a multi-party democratic process has had an influence on agenda setting. Having to win popular elections every 4 years and retain the mandate to rule have shaped the government health agenda over time. Political actors want to be seen as responsive to social values, institutional and economic challenges, societal, public and international pressures, and as interested to direct new policies and reinforce or remove existing ones.

### Content and issues framing

Policy content and ideas referred to proposed interventions to resolve problems, empirical evidence supporting a proposed solution or issue, implemented policies and policy intent as stated in documents such as laws and regulations.

The process of issue framing involves ways in which actors interpret, define and discuss policy content and ideas and surrounding conditions to take and influence institutional and political decisions as well as public opinion [3, 14]. Framing of issues ranged from people’s views and understanding of the policy content and ideas, implementation arrangements to perceptions of professionals, and the political and social implications of the policies. The framing processes linked multiple interpretations of issues to the political, social and economic contexts in which they occurred. Public and bureaucratic domains were used for discussions, debates and rebuttals in issues framing. The public domain included media platforms, while the bureaucratic domain included national level institutionalised policy-making fora such as the health summits and business meetings. In both domains, actors and stakeholders, by referring to financial, global, governmental and public interest arguments, explained their various interpretations of policy content and ideas to drive opinions on issues while trying to gain and mobilise supporters.

### Actors and power

Actors included policy agenda influencers, policy formulators and implementers who influenced the policy issue framing processes. These actors used their power derived from sources such as financial means, knowledge and access to empirical evidence and political power to move discussions and decisions in specific directions in both public and bureaucratic domains. Policy actors were fluid in their roles acting at various times as policy agenda influencers, formulators or implementers. Policy agenda influencers can broadly be grouped as (1) agenda directors, such as Presidents of Ghana, who wield political power to set a policy agenda, (2) agenda approvers, such as Ministers of Health, with political and administrative authority to approve or remove existing policy, (3) agenda advisers, such as international donors, with financial means and ideas to reshape policy discourse, and (4) agenda advocates, such as professional bodies, with campaigning skills and voice to promote issues for their benefit. Policy formulators included bureaucrats of the MOH and other government institutions with technical expertise, skills and authority to design policy content. Policy implementers were health service providers with professional expertise and discretional power to frame government policies into practice. Donors, such as the United Nations Population Fund (UNFPA), United States Agency for International Development (USAID), United Kingdom Department for International Development (DFID), among others, by virtue of financial support and global interest had over the years gained access and participated actively in the institutionalised policy-making processes in varied roles as agenda advisers, advocates and policy formulators. Central to the policy evolution phase are policy actors’ ability to consistently rely on power from their various sources (e.g. finances), knowledge and ability to act within a fast-changing context during the process of issue framing to influence policy content and ideas.

### Decision agenda item fate

The fate of decision agenda item resulted from the interactions of actors, context, content and issue framing in the decision agenda item evolution processes. We observed two different types of decision agenda item fate pathways at the end of any given ‘evolution-fate’ cycle, which we classified broadly as ‘static’ and ‘active’ fate pathways.

An ‘active fate pathway’ involved a clear documented decision at the end of the evolution processes that leads to an item disappearing, being reset or proceeding unchanged into programme formulation and implementation. Decision agenda items that follow this pathway had high-sustained political as well as administrative and bureaucratic institutional and public interest and financial support from government, international agencies, or both. The active fate pathway is iterative and an agenda item that has moved into an active pathway and a particular fate can be sent back into the evolution process and end up with a new fate. Essentially, at every stage, the policy process remains a ‘moving target’ [15]. Policy items that are reset have their content modified based on actualised needs, financial availabilities, and expectations usually put forward by policy agenda influencers who create sustained need for policy change through issues framing processes.

A ‘static fate pathway’ occurs when the result of an evolution process leads to an agenda item in question not moving into policy design and implementation, not because of an active decision to halt its progression but because of ‘no decision made’. The item is effectively ignored and neglected. A decision agenda item can lose its broader political and institutional relevance and reduce in prominence on the decision agenda over time with no agreed policy content. During issue framing, policy agenda advisers and advocates intuitively recognise this potential ‘static fate pathway’ and those actively seeking to generate interest in and support for the issue constantly seek and re-seek political, financial and institutional support.

Of our three sub-units of analysis, the ‘active’ pathway is illustrated by the maternal fee exemption policies and primary care maternal health capitation policy and the ‘static’ pathway is illustrated by free family planning as part of the NHIS policy. The next section of this paper further illustrates these three sub-units.

## Illustrations from the case studies

### ‘Static fate pathway’: Free family planning as part of the National Health Insurance Scheme (NHIS) Policy

The ‘static fate pathway’ is illustrated with the decision agenda item free family planning as part of NHIS policy [16]. Contextually, over the years, the family planning programme in Ghana has been highly subsidised by government and donors such that clients pay an out of pocket token fee averaging only approximately 10% of the international price at point of service [17]. Subsidised public health programmes such as family planning are not covered by the NHIS. Between 2008 and 2012, policy actors, such as MOH and Ghana Health Service (GHS) bureaucrats, and country representatives of international donors such as DFID, UNFPA and USAID, at various institutionalised policy dialogue for a, advocated for complete elimination of out-of-pocket payments for family planning services and its inclusion on the NHIS benefit package [18–21]. To push the ‘free family planning as part of NHIS’ agenda, the supporting MOH and GHS bureaucrats and donors considered two main points as challenges to family planning’s contribution to maternal healthcare. These were inadequate budgetary allocation and disbursement for family planning and exclusion of family planning services from the NHIS benefits package. In April 2008, the MOH and donors (Royal Danish Embassy, Royal Netherlands Embassy, DFID, European Commission, World Bank, UNFPA, WHO, United Nations International Children’s Emergency Fund (UNICEF), Embassy of Japan, USAID) discussed the 2007 health sector review findings that highlighted family planning budgetary disbursements and allocation gaps [18]. According to the review, government and donors each allocated US$1.5 million in 2007 to procure contraceptives; however, only US$1 million was disbursed (actual disbursement by each was not stated). The review also noted contraceptives supplies to the sector did not meet expected demands due to the huge and significant funding gap [22]. To address the funding gap challenges, the MOH and donors agreed on the following activities. First, the MOH was to prepare a paper on the need for increased government funding and negotiate with the Ministry of Finance and Economic Planning. Second, donors were to explore and mobilise additional funds. Finally, a cost effectiveness analysis of financing family planning services under the NHIS was commissioned [23].

Subsequently, at the July 2012 London Family Planning Summit, Ghanaian delegates issued a communique stating: “*Ghana is committed to making family planning free in the public sector and supporting the private sector to provide services...*” [24]. To follow-up on Ghana’s commitment to free family planning in the public sector, a legal backing was provided under the revised National Health Insurance Act 852. The Act 852 gazette November 2012, under the benefit section (1) stated that: “*the Minister shall prescribe the healthcare benefits package including any relevant family planning package to be provided under the National Health Insurance Scheme*” [16]. After 2012, family planning issue framing through discussions and decisions failed to move this policy decision into guidelines and implementation. The policy moved into a static pathway of ‘no decision made’ because policy agenda influencers were not able to sustain the much needed institutional and political interest and financial support. A decision was not taken to eliminate or drop the item, but a decision was not taken to move forward in any way on the item either.

The idea of ‘free family planning as part of NHIS’ was not openly contested by policy formulators, such as the MOH and GHS bureaucrats, and policy advocates, such as international donors (e.g. DFID and USAID), during health sector discussions. However, the focus of concern shifted to how the National Health Insurance Authority (NHIA), the payer agency, could reimburse providers and ensure access to contraceptives in the light of its increasing financial difficulties [25–27]. There were no answers.

“*At the health summits and during other discussions no one was against making free family planning as part of the NHIS... the major concern was how to fund its implementation*” (Policy formulator, 14/01/2014).

Contextually, since 2009, NHIA expenditure had exceeded income [28]. This contributed to delays in claims payment and refusal of services to NHIS cardholders by providers because of delayed payment [29].

“*The NHIS is saddled with unpaid claims therefore including family planning services will further over burden the scheme and compromise contraceptive access*” (Policy formulator 26/11/2012).

Putting implementation costs into perspective, the NHIA estimated that US$12,945,845.24 would be required to provide family planning as a benefit package in the year 2013, rising to US$17,016,932.02 by 2015. It was not clear how this bill was going to be picked up. The NHIA concerns were informed by the absence of government budget disbursement and the fact that funds from government and donors (mainly USAID, DFID, UNFPA, and World Bank) traditionally used to procure contraceptives were not disbursed to the NHIA [30].

In addition to the funding uncertainties and NHIA diminishing institutional interest, the policy agenda advisers and advocates, such as MOH and GHS, and donors (e.g. USAID, UNFPA, DFID) were grappling with what to include in the policy content during health sector discussions. Months on, no agreement was reached because they struggled to persuade each other and build consensus (Interview: Donor 27/11/2012). The contestation was related to how to ensure that the ‘free’ contraceptives under the NHIS were regulated, controlled and not end up on the ‘open’ market. Policy formulators such as the GHS and MOH were concerned that use of donated contraceptive commodities such as foaming tablets, pills and condoms would be difficult to monitor. Therefore, there was a need for well-defined implementing guidelines if family planning services were to be given ‘free’ in public health facilities. As noted by a policy formulator with a possible implementation constraint viewpoint: “*Family planning programme involves contraceptives; implementing a free family planning policy will present a challenge if a clear and agreed guidelines on how to implement is not developed. One needs to make sure that the contraceptives are not taken out of the facilities for private use*” (Interview: 26/11/2012). Over time, ideas on how to implement the policy became inconclusive and there was no consensus among those involved.

Additionally, political support also diminished over time. A Minister of Health put the policy item on the decision agenda in 2012 after intense lobbying by the MOH and GHS bureaucrats and donors. However, this Minister was replaced in January 2013 after a ministerial reshuffle. The agenda advisors and advocates who lobbied earlier and presented evidence that Ghana could save lives and money if family planning was financed through NHIS [17] could not sustain the political support and interest in the face of these transitions. Subsequent Ministers with authority and ability to prioritise, allocate and disburse funds were not completely mobilised and supportive of the policy. As noted by a policy formulator: “*the family planning implementation modalities discussion may be ongoing; however, I believe the energy is low. There are many other issues on the table now and this current Minister may have other priorities*” (Interview: 14/01/2014).

Therefore, although Act 852 of Parliament legally endorsed free family planning as part of the NHIS, health sector discussions around the issue ceased.

### ‘Active’ fate pathway: exclusion of primary care maternal health services from per capita provider payment

The active pathway is illustrated with the primary care maternal health services capitation policy. The policy item disappeared from the decision agenda after less than 3 months into pilot implementation of per capita provider payments in the Ashanti region because of an active decision to remove primary care maternal health services from the basket of service. The Minister of Health removed primary care maternal health services due to contestation in the public domain. There was high level government interest on the issue and concerns about the political repercussions if responsiveness was not shown in the face of the social and political discourse, as well as acrimony following pilot implementation in the Ashanti region prior to a national scale up.

The implementation moved from consensus building and resolution of conflict at a bureaucratic level into open public conflict and contestation, with wide spread media engagement and reportage in the context of an election year. During issue framing, policy implementers, service providers (particularly the Society of Private Medical and Dental Practitioners (SPMDP)) and maternity home operators questioned and opposed the technical modalities of the policy even before implementation started. As noted by a private service provider representative: “*First and foremost in principle we are not against capitation, why won’t I take money if I am paid upfront? Our main contention was the way it was implemented, the rate and the services included in the basket of services*” (Interview: 07/11/2012). The service providers’ major concerns were a low per capita rate, small numbers of enrolled subscribers and inclusion of maternity services in the basket of services. They noted that the proposed per capita rate, coupled with small numbers of enrolled subscribers, would collapse healthcare service provision. By 31st December 2011 just before policy implementation, the average number of voluntary enrolled subscribers with service providers was 46%. This was below a required NHIA target of 80% before subscribers who had not voluntarily chosen a preferred primary care provider would be administratively assigned. Given that per capita payment is based on the number of enrolees, providers with small numbers of enrolees stood to lose. The SPMDP therefore recommended new rates 15 to 20 times higher than those proposed by NHIA in a petition dated October 5, 2011, to the NHIA chief executive officer.

In addition to the low capita and enrolment rates, the private service providers argued that maternal healthcare was a national priority and should not be reimbursed through a capitation payment system because of a potential incentive for providers to reduce service inputs. This could compromise the MDG-related target of reducing the maternal mortality rate from an estimated 451 per 100,000 live births in 2008 to 185 by 2015 [31]. Service providers’ argued that the policy could derail maternal health outcome gains. As noted by a service provider: “*Maternal services should not have been added in the first place, this can derail the maternal health outcome achievements made so far. Under capitation there is a high incentive for providers to reduce inputs for service delivery*” (Interview: 04/09/2012).

After a back and forth process of discussions in the bureaucratic arena on the technical contents and implementation challenges between the NHIA and service providers, a consensus was not reached on which services to exclude and a possible increase in the per capita payment. The SPMDP and maternity home representatives, after failing to reach a consensus with the NHIA, moved discussions and their interpretations of the policy into the public arena using media platforms to draw others into the discussions and mobilise support for their recommendations and framing of the policy technical content [32, 33]. The private service providers had power and mobilised support, drawing on public respect, social and professional identity, and access to other influencers such as NHIS enrolees. “*The private service providers were all over the radio and TV stations discussing capitation policy and how the NHIA is imposing the policy on them...*” (Public service provider, 04/09/2012).

The SPMDP and maternity home representatives quickly attracted social and political support from the general public, a mobilised pressure group, health professional bodies, and opposition politicians. These organised supporters all focused on different aspects of the capitation policy in the process of issue framing on media platforms. For example, a mobilised pressure group, the Ashanti Development Union, focused on the political context within which the policy was implemented and questioned the rationale for piloting such a policy in the Ashanti region. “*Some people believe this is political, this is to punish the region for voting against the government in power*” (GHS staff, 28/8/2012). They argued that the policy implementation may be politically motivated and should be viewed as a punishment since the region had traditionally voted against the current government [34]. Moreover, in an election year (as was 2012), such arguments seemed appropriate to attract the government’s response. The Pharmaceutical Society of Ghana and Ghana Medical Association focused on the technical content and consensus building and recommended implementation postponement for more education and until all stakeholders agree on the capitation services and rates [35–37]. On the other hand, opposition politicians and the general public took to the streets to show their solidarity and supported calls for the suspension of the policy [38, 39].

Amid increased social and political support from the general public, professional bodies and opposition politicians as well as the lack of consensus on the policy modalities, the SPMDP and maternity homes suspended their services to NHIS card holders. They stated in a press release that the NHIA had imposed the policy to the detriment of quality care and health facilities [40]. The private clinics and maternity homes operated approximately 53% of health facilities in the region [41]. Therefore, suspension of their services to NHIS card holders was a major setback to the government’s intervention to remove out-of-pocket payments at the point of use and increase financial access to healthcare through a social health insurance scheme [42].

The ongoing intense process of issue framing on the media platforms, potential effect on maternal health outcomes and the fact that NHIS cardholders will have to pay out-of-pocket for healthcare services in NHIA-accredited private health facilities attracted the attention of the President and the Minister of Health [43, 44]. In the context of parliamentary and presidential elections due in December 2012, the government stood to gain political points by listening and responding favourably to the opponents of the policy implementation. Therefore, the Minister of Health, in consultation with the NHIA and service providers, removed the primary care maternal health service capitation policy from the decision agenda [45].

### ‘Active’ fate pathway: maternal health fee exemption policy

The active pathway is illustrated with maternal health fee exemption policies. At the time of the study, the maternal health fee exemption policy had been periodically reset eight times over the five decades since first introduced in 1963. Table 4 summarises these policy contents and reset category. Diverse policy agenda influencers, policy formulators and implementers in various national and international contexts characterised by high sustained political and institutional interest had periodically framed the policy item.

**Table 4:** Summary of policy contents and reset category for maternal health fee exemption between 1969 and 2012

Reset category	Year	Policy Content
Policy Expansion	1969	“*All antenatal services provided at Government hospitals should be for free*”
	1971	“*No fees other than the fees prescribed for accommodation and maintenance shall be paid in respect of services rendered in a hospital to* *- (b) any person other than a non- resident alien in respect of antenatal care at a health post, rural health centre or clinic, or any other hospital specified by the Director of Medical Services by notice published in the Gazette;* *- (c) any maternity patient who has had four or more child births;* *- (d) any maternity patient referred to a hospital from a clinic or health centre;* *- (e) any maternity patient referred to a hospital by a registered midwife or registered medical practitioner*”
Policy Contraction	1983	“*No fees other than hospital accommodations and catering services shall be paid in any Government hospital or clinic in respect of* *– (i) antenatal and postnatal services*”
	1985	“*No fees other than hospital accommodations and catering services shall be paid in any Government hospital or clinic in respect of* *– (i) antenatal and post-natal services*”
	1997	“*Exemption for antenatal services (first four antenatal care visits) in government health facilities*”
Policy Expansion	2003	“*User fee exemption for maternal services in Northern, Upper-West, Upper-East and Central Regions in government, private and mission health facilities*”
	2005	“*User fee exemption for maternal services in all ten regions in government, private and mission health facilities*”
	2008 & 2012	“*National Health Insurance Scheme premium exemption for all pregnant women in Ghana*” Benefit under the free maternal care policy • No premium for fresh registration or renewal of membership • No processing fee for registration or renewal • Antenatal period: free antenatal, general services and medicines • Delivery: free services and medicines, including caesarean • Postnatal period: free services and medicines • Full year cover no matter when pregnant woman registers • Free care for the baby on mother’s National Health Insurance Scheme ticket for 90 days • Alternatively, the baby can be treated free on the father or other designated guardian • After 90 days the child can be registered as an individual under 18 (no premium but processing fee required)

During the 1960s and 1970s, the fee exemption for maternal services policy item evolved through policy content expansion. Contextually, changes in government, high maternal deaths and social unrest mainly informed the policy framing processes. After the overthrow of Dr Nkrumah (Ghana’s first president) in a 1966 coup by the National Liberation Council, the military government blacklisted all existing national social policies for political and economic reasons. The military government decided to reintroduce hospital fees, a decision which led to public unrest [46–48]. However, this did not deter the government from designing the Hospital Fees Decree. During the design process, MOH bureaucrats advocated and expanded the existing maternal health fee exemption policy of free antenatal services at government hospitals to include delivery services for multiparous patients because of high maternal deaths (Interview: Former MOH Staff 15/07/2013).

The proposed Hospital Fees Decree was suspended by a new civilian government that took power on the same date that the Decree was to be implemented on October 1, 1969. Therefore, the existing minimal hospital fees and fee exemption for antenatal services continued [46, 49]. However, due to a further declining economy [50] and increasing healthcare expenditures, the Busia government in 1971 introduced a Hospital Fees Act [51]. However, as noted by a former MOH staff, maternal health fee exemptions for antenatal and multiparous patients were maintained because of increasing maternal deaths (Interview: 15/07/2013). The subsequent military and civilian governments that followed Busia’s government after a coup in January 1972 maintained the maternal health fee exemptions and minimal charging because it was popular with the general populace [52, 53].

After a series of political changes between 1972 and 1980, the Provisional National Defence Council (PNDC) came to power through a coup in 1981, paving the way for another opportunity to frame the maternal fee exemption policy. Between 1983 and 1997, the maternal fee exemption policy evolved through policy content contraction during a hospital fee regulation design. Contextually, economic decline and international influence were the main drivers of the hospital fee regulation initiative. Gross domestic product per capita fell from US$281 in 1970 to US$180 in 1983 and state institutions and public services were gravely damaged and under-resourced [54]. The decline in the health budget led to a reduced capacity to procure medicines and consumables (Interview: Former MOH Staff 15/07/2013).

The MOH bureaucrats designed the hospital fees regulation with approval from the PNDC government to charge fees and generate funds to prevent further decline of public healthcare service provision [55]. However, UNICEF, an international policy agenda advocate contested the absence of fee exemption for maternal health services. As noted by a former MOH staff: “*The first time we introduced the regulations, UNICEF was against our fees because for them the policy is that maternal care should be free...*” (Interview: 22/08/2012). Fee exemptions for antenatal and postnatal services were therefore included in the 1983 hospital fees regulation. In 1985, the hospital charges were increased to fully recover healthcare costs [56], but fee exemptions for antenatal and postnatal services were maintained because, over time, it had become a safe net for the poor (Interview: MOH Staff 27/09/2012).

A change in the political scene and government financial inflows to the health sector facilitated another maternal health fee exemption policy agenda reset. The PNDC reorganised itself into a political party, the National Democratic Congress, and won multiparty elections in December 1992 and 1996. In January 1997, the President gave a directive to include delivery services to the existing antenatal and postnatal fee exemption policy within a context of high maternal deaths and reduced supervised delivery attendance in health facilities [57–59]. In framing the President’s directive, the MOH bureaucrats considered the insufficient financial inflows from the government into the health sector. Based on their experience and judgement of what was feasible and practical to implement, they narrowed the existing maternal health fee exemption policy to only four antenatal visits in November 1997 (Interview: MOH Staff 27/09/2012).

In the 2000s, changes in government, maternal deaths and international health agendas related to the Heavily Indebted Poor Countries (HIPC) initiative and MDGs necessitated policy issue framing processes. The National Democratic Congress lost the December 2000 election and the New Patriotic Party came to power. The New Patriotic Party government was faced with stagnant and even regressive economic growth and, in 2001, opted for debt relief under the HIPC initiative of the International Monetary Fund and Work Bank [47, 60]. To benefit from the HIPC initiative, Ghana developed a poverty reduction strategy directed towards attainment of anti-poverty objectives consistent with the MDGs [61]. The process of aligning to the poverty reduction strategy created the opportunity to reset the existing maternal fee exemption policy agenda to benefit the relatively poorer and deprived regions. Thus, to reflect national priorities, the policy to provide free antenatal, delivery and postnatal services in all government, private and mission facilities in the four most deprived regions (northern, upper-west, upper-east and central) was made [62].

High maternal deaths in non-deprived regions [63] presented an opportunity for MOH bureaucrats, donors and other stakeholders during health sector discussions to frame the policy item in 2004. During the health summit meetings in December 2004, the MOH and stakeholders argued that a national maternal mortality rate of 503 per 100,000 live births was high and there were pockets of extreme poverty across the country and not only in the regions labelled as deprived. A proposal was therefore made to extend the fee exemption for antenatal, delivery and postnatal services to ‘non-deprived’ regions to help reduce maternal mortality [63]. The government in 2005 approved the proposal [64].

By 2007, health facilities had stopped implementing the policy because of mounting unpaid bills and limited financial resources for reimbursement from the MOH [65]. The MOH bureaucrats, donors and other stakeholders during the April 2008 health summit discussed how the suspended maternal health fee exemption policy had contributed to worsening maternal health outcomes. The discussion was based on empirical evidence of a decreased proportion of supervised deliveries from 44.5% in 2006 to 35.1% in 2007 and an increased institutional maternal mortality ratio from 187/100,000 live births in 2006 to 224/100,000 in 2007 [18].

The government intervened and announced a free maternal care policy in May 2008. DFID health sector budget support was allocated by the government to implement the policy (Interview: Former MOH Staff 5/11/2012). Policy formulators at the MOH decided to implement the free maternal care directive through an insurance premium fee exemption for pregnant women. This was against the backdrop of previous implementation challenges, secured funding and lobbying by the NHIA to implement the policy [65, 66]. The NHIS premium fee exemption policy for all pregnant women in Ghana included antenatal, delivery and postnatal services and is stated in the 2012 National Health Insurance Act 852 [16].

## Discussion

Our explanatory framework of how and why health policy items in Ghana fared once they got onto the decision agenda shows that the dynamics are complex. Political and institutional arrangements, government responsiveness, financial availability, international agendas, empirical evidence and changing issue framing periodically lead to decision-making cycles in relation to a given agenda item. Each cycle ends with a decision (active pathway) or non-decision (static pathway) of the item, which is not an end point. An item which ended in a ‘static’ or ‘active’ pathway in a given process cycle may enter a new decision-making cycle, and as a result of a new cycle end up in a different or the same but modified fate at the end of the next process. Varied policy actors, such as bureaucrats, international donors, health professional bodies and the general public with power, ideas and problem definitions within constantly changing political and socioeconomic context shaped maternal health policy item evolution and fate on the decision agenda in Ghana.

The maternal health agenda item evolution and fate framework reflects, but also builds upon, some of the already existing policy analysis frameworks in the literature. First is the Walt and Gilson [13] policy triangle, which explains policy processes and outcomes in terms of context, content, process and actors, rather than policy content alone. As can be seen from Fig. 2, context such as change in government, content in the form of policy intent, actors such as policy agenda influencers, and multiple interpretations of issues were central in understanding the evolution of items on the decision agenda. The inter-relationships and dynamics around policy content and ideas, national and international context, and varied actors and their power were critical influences on the constant process of issue framing that shaped policy item evolution on decision agenda over time.

Second, is Grindle and Thomas’s conceptualisation of policy change in public and bureaucratic arenas [67]. As in the maternal health agenda evolution and fate framework, politics and institutional structures also determined the venues in which issue framing occurred. The public and bureaucratic institutional venues were interrelated and fluid in that a specific issue framing and decision did take place in more than one venue. Additionally, framing processes in the public venues generally had political influence and effect decisions and actions within the institutionalised dialogue fora. Expert recommendations from institutionalised dialogues also generated discussions on media platforms and public unrest. Issue framing venues in public and bureaucratic domains became politically relevant and critical to maternal health policy item evolution and fate when policy actors linked different responses and actions of politicians, bureaucrats, international donors, service providers, professional bodies and the general public in a complex and unpredictable manner. This gave basis for multiple interpretation of actions, inactions and motives of other policy actors.

The institutional structures within which maternal health issues framing occurred set limits on who participated in the decision-making processes, as well as the level of influence among the policy actors and how they interpreted ideas and evidence. In the case of the ‘free family planning as part of NHIS’ policy item, the policy agenda advocates and advisers operated in ‘closed venues’ within the bureaucratic institutionalised policy dialogues as they framed issues. The MOH and GHS bureaucrats, international donors such as DFID, UNFPA, service providers and Ministers of Health responded differently to the evidence of potential financial gains. As noted by Portz [14], policy actors do present different explanations for the nature and solution of a particular problem; in our case, the impact of their collective influence of different and diverse understandings of how to implement such a policy failed to move the policy beyond the decision agenda. Again, within the ‘closed venue’ structure, health professional groups and the general public with lobbying skills and social capital were not actively involved in the framing discussions nor were they mobilised to contribute to such discussions. The framing processes and the institutional context within which discussions and decisions occurred is similar to decision-making processes within bureaucratic arenas and politics as usual scenarios described by Grindle and Thomas [67].

However, in the case of the primary care maternal health service capitation policy, a broader section of policy agenda influencers, policy formulators and implementers were involved in the issue framing. Technical discussions on the benefit package and reimbursement rates were moved from the bureaucratic institutional structures into the public domain and subjected to multiple and divergent interpretations on the media platforms. Within the context of Ghana’s democracy and free speech, the private service providers, organised groups and the general public were able to move the issue framing in specific directions [12]. In the political context of an election year, the President and Minister of Health responded favourably to the demands made in the public domain; as argued by Stone [68], political actors respond to multiple interpretations of government policies to control contestations and conflict. The collective actions and inactions of the varied policy actors through government responsiveness, NHIA disagreement with service providers, multiple interpretations of the policy content on the media platforms, social mobilisation of the general public and professional bodies as a network of influence, created a crisis situation. The crisis situation created by the actions, inactions and motives of the diverse policy actors is similar to the perceived crisis scenario of high politics with broad categories of support groups described by Grindle and Thomas [67].

In the case of maternal fee exemption, the policy item evolved and reset through policy content expansion, contraction and expansion on the decision agenda over five decades. The framing processes linked to the policy reset initially evolved from bureaucratic institutional structures and public domains to only bureaucratic institutional structures. Actions in these venues collectively shaped the policy content over time in diverse political, economic and social contexts. The maternal fee exemption policy was periodically modified to meet changing needs and expectations of policy agenda directors, approvers, advisers and advocates who constantly promoted policy change. The framing processes that occurred over years did so within an environment of sustained political and institutional interest and financial support.

Surrounding situations that prompted maternal health issue framing varied within the public and bureaucratic domains though there were common triggers such as changes in government and election year. Surrounding situations that prompted maternal health issue framing in the public domain included the general public’s perceived effect of government policy and anticipated risk of policy implementation, societal political leaning and administrative challenges such as unresolved negotiations between policy-makers and implementers. Surrounding situations that prompted maternal health issue framing in the bureaucratic institutional structures included maternal health-related empirical evidence from routine health management information systems, demographic health surveys, cost effectiveness analysis, economic and financial analysis, international and national agendas, financial availability, existing policy implementation challenges, and interventions proven to improve maternal health outcomes.

The maternal health agenda evolution and fate framework also underlines the importance of financial resources, agreeable ideas, and political support and responsiveness as the principal solution to maternal health problems. Therefore, those with the financial resources and able to push an agreeable idea as well as those with political influence, or access to it, are particularly powerful during maternal health policy issue framing and are able to mobilise support to move issues beyond the decision agenda. As suggested by Rochefort and Cobb [3], an issue framing process with a solution is likely to garner general support. Such ‘solutions’ dominate the issue framing process as powerful actors are able to persuade others to see the ‘solutions’ in their policy narratives.

In sum, the framework and assumed relationship between content and ideas, actors and their powers, context and issue framing provide an understanding of how policy actors within a changing context used their power sources to define maternal health problems and the frame accompanying a course of action in the Ghanaian health sector. The framework’s strength lies in its theoretical insights. One is how bureaucrats, donors, service providers and political actors within a LMIC setting were able to exercise power and maintain access to other stakeholders under different periods and situations to influence and make decisions with respect to maternal health policy agenda. The second strength is the categorisation of policy actors based on their actions as policy agenda directors and approvers (those who take or approve final decisions) and agenda advisers and advocates (those who influence decisions). A third insight is how bureaucratic and public venues present different policy actors with unique opportunities to interact and use their power sources and surrounding situations as a negotiation tool to influence decisions.

The framework also has its limitations. First, the theoretical insights were based on maternal health policies in Ghana and are not generalisable to other health priorities and sectors or other LMICs. Second, the framework, in its current form, does not take into account maternal health policy agenda items that were resolved quickly and moved into formulation and implementation. Beyond these limitations, the framework can be put forward for consideration and testing in other health priorities and sectors to test replication logic. The theoretical insights can be tested through comparative case studies of maternal health policy agenda items across LMIC settings.

## Conclusions

Given the complexity of policy processes, the maternal health agenda evolution and fate framework presents policy evolution drivers in terms of policy content and ideas, context, actors and their powers, and issue framing within both bureaucratic and public venues to better understand the policy process. Our study highlights the interconnectedness of these drivers in shaping policy items evolution through ‘active’ and ‘static’ pathways. Change agents need to understand and work within context, stakeholder interest, power, ideas and framing of issues, making room for a certain amount of shifting evolution over long rather than short periods of time. Moreover, the influence of an actor’s power on the policy process depends on the actor’s efforts and ability to use power to mobilise and convince final decision-makers to move issues in specific directions. Understanding policy processes is relevant across other LMICs. We hope this framework contributes to policy analysis and learning in Ghana and beyond.

## Abbreviations

DFID: United Kingdom Department for International Development
GHS: Ghana Health Service
HIPC: Heavily Indebted Poor Countries initiative
LMIC: low- and middle-income countries
MDG: Millennium Development Goal
MOH: Ministry of Health
NHIA: National Health Insurance Authority
NHIS: National Health Insurance Scheme
PNDC: Provisional National Defence Council
SPMDP: Society of Private Medical and Dental Practitioners
UNFPA: United Nations Population Fund
UNICEF: United Nations International Children’s Emergency Fund
USAID: United States Agency for International Development
